# Exploring subdomain variation in biomedical language

**DOI:** 10.1186/1471-2105-12-212

**Published:** 2011-05-27

**Authors:** Thomas Lippincott, Diarmuid Ó Séaghdha, Anna Korhonen

**Affiliations:** 1 Computer Laboratory, University of Cambridge, 15 JJ Thomson Avenue, Cambridge CB3 0FD, UK

## Abstract

**Background:**

Applications of Natural Language Processing (NLP) technology to biomedical texts have generated significant interest in recent years. In this paper we identify and investigate the phenomenon of linguistic *subdomain variation *within the biomedical domain, i.e., the extent to which different subject areas of biomedicine are characterised by different linguistic behaviour. While variation at a coarser domain level such as between newswire and biomedical text is well-studied and known to affect the portability of NLP systems, we are the first to conduct an extensive investigation into more fine-grained levels of variation.

**Results:**

Using the large OpenPMC text corpus, which spans the many subdomains of biomedicine, we investigate variation across a number of lexical, syntactic, semantic and discourse-related dimensions. These dimensions are chosen for their relevance to the performance of NLP systems. We use clustering techniques to analyse commonalities and distinctions among the subdomains.

**Conclusions:**

We find that while patterns of inter-subdomain variation differ somewhat from one feature set to another, robust clusters can be identified that correspond to intuitive distinctions such as that between clinical and laboratory subjects. In particular, subdomains relating to genetics and molecular biology, which are the most common sources of material for training and evaluating biomedical NLP tools, are not representative of all biomedical subdomains. We conclude that an awareness of subdomain variation is important when considering the practical use of language processing applications by biomedical researchers.

## Background

### Overview of biomedical natural language processing

Research in the field of NLP is concerned with the development of systems that take textual data (e.g., research articles or abstracts) as input and/or output. Examples of these systems encompass "core" tasks that often provide components of larger systems, such as syntactic analysis or semantic disambiguation, as well as practical applications for tasks such as summarisation, information extraction and translation. Over the past decade, the new field of biomedical text processing has seen dramatic progress in the deployment of NLP technology to meet the information retrieval and extraction needs of biologists and biomedical professionals. Increasingly sophisticated systems for both core tasks and applications are being introduced through academic venues such as the annual BioNLP workshops [1] and also in the commercial marketplace.

This meeting of fields has proven mutually beneficial: biologists more than ever rely on automated tools to help them cope with the exponentially expanding body of publications in their field, while NLP researchers have been spurred to address important new problems in theirs. Among the fundamental advances from the NLP perspective has been the realisation that tools which perform well on textual data from one source may fail to do so on another unless they are tailored to the new source in some way. This has led to significant interest in the idea of contrasting *domains *and the concomitant problem of domain adaptation, as well as the production of manually annotated domain-specific corpora.

In this paper we study the phenomenon of *subdomain variation*, i.e., the ways in which language use differs in different subareas of a broad domain such as "biomedicine". Using a large corpus of biomedical articles, we demonstrate that observable linguistic variation does occur across biomedical subdisciplines.

Furthermore, the dimensions of variation that we identify cover a wide range of features that directly affect NLP applications; these correspond to variation on the levels of lexicon, semantics, syntax and discourse. In the remainder of this section - before moving on to describe our methods and results - we motivate our work by summarising related prior research on corpus-based analysis of domain and subdomain variation and on the recognised problem of domain adaptation in natural language processing.

### Analysis of subdomain corpora

The notion of *domain *relates to the concepts of *topic*, *register *and *genre *that have long been studied in corpus linguistics [2]. In the field of biomedical NLP, researchers are most often concerned with the genre of "biomedical research articles". There is also a long history of NLP research on clinical documentation, frequently with a focus on extracting structured information from free-text notes written by medical practitioners [3-6].

A number of researchers have explored the differences between non-technical and scientific language. Biber and Gray [7] describe two distinctive syntactic characteristics of academic writing which set it apart from general English. Firstly, in academic writing additional information is most commonly integrated by modification of phrases rather than by the addition of extra clauses. For example, academic text may use the formulations *the participant perspective *and *facilities for waste treatment *where general-audience writing would be more likely to use *the perspective that considers the participant's point of view *and *facilities that have been developed to treat waste*. Secondly, academic writing places greater demands on the reader by omitting non-essential information, through the frequent use of passivisation, nominalisation and noun compounding. Biber and Gray also show that these tendencies towards "less elaborate and less explicit" language have become more pronounced in recent history.

We now turn to corpus studies that focus on biomedical writing. Verspoor et al. [8] use measurements of lexical and structural variation to demonstrate that Open Access and subscription-based journal articles in a specific domain (mouse genomics) are sufficiently similar that research on the former can be taken as representative of the latter. While their primary goal is different from ours and they do not consider variation across multiple different domains, they do compare their mouse genomics corpus with small reference corpora drawn from newswire and general biomedical sources. This analysis unsurprisingly finds differences between the domain and newswire corpora across many linguistic dimensions; more interestingly for our purposes, the comparison of domain text to the broader biomedical superdomain shows a more complex picture with similarities in some aspects (e.g., passivisation and negation) and dissimilarities in others (e.g., sentence length, semantic features). Friedman et al. [9] document the "sublanguages" associated with two biomedical domains: clinical reports and molecular biology articles. They set out restricted ontologies and frequent co-occurrence templates for the two domains and discuss the similarities and differences between them, but they do not perform any quantitative analysis. Hirschman and Sager [3] document aspects of clinical writing that affect language processing systems, such as a pronounced tendency towards ellipsis and the use of phrases in the place of full sentences; similar observations are made in the corpus study of Allvin et al. [10].

Other researchers have focused on specific phenomena, rather than cataloguing a broad scope of variation. Cohen et al. [11] carry out a detailed analysis of argument realisation with respect to verbs and nominalisations, using the GENIA and PennBioIE corpora. Nguyen and Kim [12] compare the behaviour of anaphoric pronouns in newswire and biomedical corpora; among their findings are that no gendered pronouns (such as *he *or *she*) are used in GENIA while demonstrative pronouns (such as *this *and *that*) are used far more frequently than in newswire language. Nguyen and Kim improve the performance of a pronoun resolver by incorporating their observations, thus demonstrating the importance of capturing domain-specific phenomena.

### Domain effects in Natural Language Processing

Recent years have seen an increased research interest in the effect of domain variation on the effectiveness of Natural Language Processing (NLP) technology. The most common paradigm for implementing NLP systems (whether in a biomedical or general context) is *statistical *or *machine learning*, whereby a system learns to make predictions by generalising over a collection of *training data *(e.g., a set of documents) that has been annotated with the correct output. A fundamental assumption of statistical methods is that the data used to train a system has the same distribution as the data that will be used when applying or evaluating the system. When this assumption is violated there is no guarantee that performance will generalise well from that observed on the training data and in practice a decrease in performance is usually observed. The dimensions of variation that directly affect a given statistical tool will depend on the application and methodology involved. For example, a document classifier using a bag-of-words representation will be sensitive to lexical variation but not to syntactic variation, while a lexicalised parser will be sensitive to both.

In many NLP tasks, a standard set of human-annotated data is used to evaluate and compare systems. These data sets are often drawn from a single register or topical domain, news text being the most common due to its availability in large quantities. For example, syntactic parsers are usually trained and evaluated on the Wall Street Journal portion of the Penn Treebank, though it is known that this gives an overoptimistic view of parser accuracy [13,14]. For example, Gildea [13] demonstrates that a parser trained on the Wall Street Journal section of the Penn Treebank suffers a significant drop in accuracy when tested on the Brown corpus section of the Treebank, which is composed of general American English text. Losses in performance caused by mismatch between training and test domains have been observed for a wide range of problems, from sentiment classification of reviews about different classes of products [15] to named entity tagging for printed and broadcast news text [16].

When considering the transfer of NLP tools and techniques to biomedical text processing applications, the distance between source and target domains is far greater than that between the Brown and WSJ corpora or between film and electronics reviews on an on-line retailer's website. As described in the previous section, the language of biomedical text differs from general language in many diverse ways, making an awareness of variation effects crucial. Two strategies are available to developers of tools for statistical biomedical text processing: creating a new annotated corpus of domain-specific data, and "adapting" a model trained on an existing out-of-domain data set to the domain of interest. The strategies are complementary: domain adaptation methods usually require some amount of annotated target-domain data, while the construction of specialised domain corpora for complex tasks is extremely labour-intensive and it is infeasible to produce large standalone corpora for multiple tasks and domains. Describing the range of methods that have been introduced by NLP and machine learning researchers for domain adaptation is beyond the scope of this paper; for a representative sample see [15-19] and the proceedings of the ACL 2010 workshop on Domain Adaptation for NLP [20].

There are many examples of corpora constructed to facilitate the implementation and evaluation of tools for specific problems in biomedical language processing, for example the BioScope corpus [21] for speculative language detection and the BioCreative I and II gene normalisation corpora [22,23]. There are also text collections that have been annotated for multiple tasks, most notably GENIA [24], PennBioIE [25] and BioInfer [26]. One common feature of these corpora is that they have been compiled from just one or two specific subject areas, typically molecular biology. GENIA consists of 2,000 abstracts dealing with transcription factors in human blood cells. PennBioIE is also a corpus of abstracts, in this case covering topics in cancer genomics and the behaviour of enzymes affecting a particular family of proteins. BioInfer contains 1,100 sentences that relate to protein-protein interactions. While these are without a doubt extremely valuable resources for application building, their limited coverage casts doubt on the assumption that a system that performs well on one will also perform well on biomedical text in general. One of the central questions addressed in the present paper is how representative a corpus restricted to a single subdomain of biomedical text can be of the overall biomedical domain.

## Methods

We now describe the implementation details of our study: first, we present the OpenPMC corpus of biomedical text and its division into the subdomains that constituted our basic units of enquiry. Second, we enumerate the linguistic features we considered, and explain how we extracted them from the corpus. Third, we describe our choice of metric for measuring divergence between subdomains, and our approach to gauging its statistical significance. Fourth, we describe the clustering method we used on the raw feature distributions. Finally, we explain how the results of these methods are presented graphically.

### Data set and preprocessing

**Figure 1:**
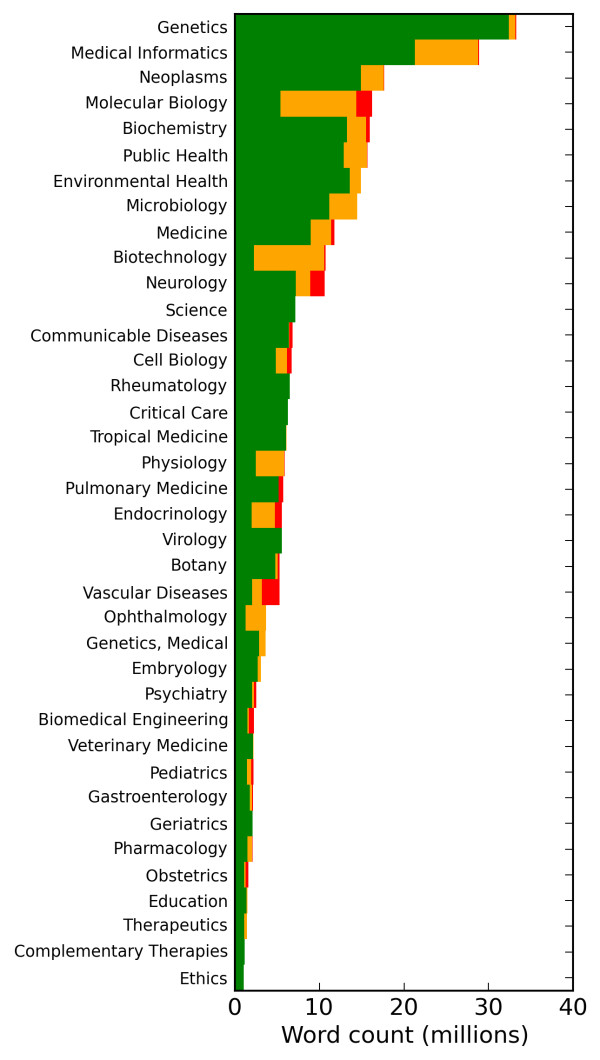
**Distribution of OpenPMC data by subdomain**. OpenPMC word count for the subdomains we consider: green coloring indicates data mapped to a single subdomain, orange indicates two subdomains, and red indicates three or more. In this study we only use data mapped to a single subdomain.

The Open Access Subset of PubMed (OpenPMC) is the largest publicly available corpus of full-text articles in the biomedical domain [27]. OpenPMC is comprised of 169,338 articles drawn from 1,233 medical journals indexed by the Medline citation database, totalling approximately 400 million words. Articles are formatted according to a standard XML tag set [28]. The National Institute of Health (NIH) maintains a one-to-many mapping from journals to 122 subdomains of biomedicine [29]. The mapping covers about a third of the OpenPMC journals, but these account for over 70% of the total data by word count. Journals are assigned up to five subdomains, with the majority assigned one (69%) or two (26%) (Figure 1). Our data set is composed of journals that are assigned a single subdomain. To ensure sufficient data for comparing a variety of linguistic features, we discarded the subdomains with less than one million words of data. This makes for a total of 342 journals in 38 biomedical subdomains. We also added a reference subdomain, "Newswire", composed of a 6 million word random sample from the Gigaword corpus. These subdomains were our initial objects of comparison.

### Feature choice motivation

We considered subdomain variation across a range of lexical, syntactic, semantic, sentential and discourse features. Here, we motivate our choices and point to NLP applications that make use of specific features, and hence are potentially affected by their variation.

#### Lexical features

Differences in vocabulary are what first come to mind when defining subdomains, and to measure this we considered lemma frequencies. A lemma is a basic word-form that abstracts beyond inflection: for example, the tokens "runs", "run" and "running" would all be considered instances of the verb lemma "run". We considered noun, verb, adjective and adverb lemma frequencies separately. Lexical features are fundamental to methods for text classification [30], language modelling [31] and most modern parsing approaches [14,32,33]. These systems may therefore be affected by variations in lexical distributions, either as a result of misestimating frequencies or out-of-vocabulary effects.

Part-of-speech (POS) tags capture lexical properties not preserved by lemmatisation, such as singular vs. plural and passive vs. active, as well as various function words. At the same time, POS tags abstract over potentially large classes of words such as the class of all common nouns. For example, "runs" may be tagged "VBZ", indicating that it is 3rd person singular, while "running" may be tagged "VBG", indicating it is a present participle. POS tags reflect several known features of scientific language, such as pronominal usage, verb tense and punctuation. POS tagging is a first step in many NLP tasks, such as morphological analysis and production [34] and constructing lexical databases [35].

#### Syntactic features

Lexical categories that describe a word's combinatorial properties are essential to the success of some classes of lexicalised statistical parser. In the framework of combinatory categorial grammar (CCG) lexical categories are the essential bridge between the lexical and syntactic levels, encoding information on how a lexical item combines with its neighbours to form syntactic structures [36]. CCG categories are assigned by a "super-tagger" sequence labeller, akin to the process for POS tags. For example, the most frequent CCG category for the verb "run" is "(S[b]\NP)/NP", which indicates it combines with a noun phrase to the right, then to the left, to form a sentence. CCG categories have been proposed as a good level for hand-annotation when re-training lexicalised parsers for new domains [19], as they provide syntactic information while remaining relatively easy for non-experts to label, compared e.g. to full sentence parse-trees. Changes in their distribution would affect parsing accuracy, but could be a tractable starting-point for domain adaptation if the problem is anticipated.

Grammatical relations (GR), also called syntactic dependencies, specify relationships between words, and by extension, between higher-level syntactic structures. For example, the two GRs "ncsubj(runs, dog)" and "dobj(runs, home)" indicates that "dog" is the subject, and "home" the object, of the verb "runs", as in the sentence "The dog runs home". More complex sentences may include multiple clauses, and so further distinctions are made between clausal and non-clausal arguments (e.g. the "nc" in "ncsubj"). GR distributions will reflect characteristic syntactic preferences in a domain, such as the preference for modification observed by Biber and Gray [7] in scientific text. Variation in GR distributions across subdomains may be expected to degrade parsing performance and necessitate model adaptation.

#### Semantic features

Semantic features capture what a text is "about" at a more general and interpretable level than individual lexical features. One approach we adopted, known as "topic modelling", models each document of interest as a mixture of distributions over words or "topics" that have been induced automatically from the corpus. These topics provided a bottom-up vocabulary for investigating semantics in the corpus that is complementary to the top-down vocabulary provided by the NIH subject headings.

We also investigated a more specific kind of semantic behaviour relating to verb-argument predication. This was motivated by the observation that relations between verbs and their arguments are central to important semantic tasks such as semantic role labelling [37,38]. Variation in the pattern of verb-argument relations across subdomains is likely to indicate difficulty in porting tools from one subdomain to another.

#### Sentential and discourse features

Sentence length is known to roughly correlate with parsing difficulty and syntactic complexity [39]. Noun phrase (NP) length increases as more information is "packed" via pre-/post-modification. Scientific language is known to aim for high information density [7,40]. Pronominal usage, which is touched on by POS tags, can be a stylistic indicator of scientific writing at a finer level, e.g. the avoidance of personal pronouns in laboratory sciences, and the restriction of gendered pronouns mainly to clinical sciences [40]. Co-reference resolution is crucial to many information extraction applications where valuable information may be linked to a referent in this fashion. Nguyen and Kim [12] compare the use of pronouns in newswire and biomedical text, using the GENIA corpus as representative of the latter, and found significant differences. Moreover, they improved the performance of a pronoun resolution system by tailoring it based on their findings, which demonstrates the practical value in considering these features.

### Feature extraction

#### Lexical and syntactic features

We first converted each OpenPMC article from XML to plain text, ignoring "non-content" elements such as tables and formulae, and split the result into sentences, aggregating the results by subdomain. The sentences were fed to the C&C parsing pipeline [41], using POS tagging and supertagging models augmented with training on the GENIA corpus of annotated biomedical texts [19]. C&C uses the *morpha *morphological analyser [34], maximum entropy labellers for tagging and supertagging and a log-linear parse model. RASP-parser-style [42] grammatical relations were extracted from C&C output using deterministic rules. Tables 1 and 2 show the system's output for the sentence "Multiple twinning in cubic crystals is represented geometrically".

**Table 1:** Feature extraction from example sentence

Lemma	POS	CCG category
multiple	JJ	N/N

twinning	NN	N

in	IN	(NP\NP)/NP

cubic	JJ	N/N

crystal	NNS	N

be	VBZ	(S[dcl]*\*NP)/(S[pss]*\*NP)

represent	VBN	S[pss]*\*NP

geometrically	RB	(S\NP)*\*(S\NP)

Lexical features extracted from the sentence "Multiple twinning in cubic crystals is represented geometrically" using the C&C parser

**Table 2:** Grammatical relations of example sentence

Grammatical relation	First argument	Second argument
ncmod	twinning	Multiple

ncmod	represented	geometrically

aux	represented	is

ncsubj	represented	twinning

Grammatical relations extracted from the sentence "Multiple twinning in cubic crystals is represented geometrically" using the C&C parser

From this output we simply counted occurrences of noun, verb, adjective and adverb lemmas, POS tags, GRs and CCG categories. The lemma distributions tended to be Zipfian in nature, while the others did not. We experimented with filtering low-frequency items at various thresholds, to reduce noise and improve processing speed, and settled on filtering items that occur less than 150 times in the entire corpus.

#### Sentential and discourse features

We measured average sentence, noun phrase and base nominal lengths (in tokens) for each subdomain, using the parsed output from C&C. In order to filter out lines that are not true sentences, we ignored lines containing less than 50% lowercase letters. Sentence length is defined as the number of non-punctuation tokens in a sentence. Noun phrase length is defined in terms of a sentence's dependency structure as the number of words from the leftmost word dominated by a head noun to the rightmost dominated word. Base noun phrase length is simply the number of tokens contained in the head noun and all premodifying tokens. As our corpus is not annotated for coreference we restricted our attention to types that are reliably coreferential: masculine/feminine personal pronouns (*he*, *she *and case variations), neuter personal pronouns (*they*, *it *and variations) and definite noun phrases with demonstrative determiners such as *this *and *that*. To filter out pleonastic pronouns we used a combination of the C&C parser's pleonasm tag and heuristics based on Lappin and Leass [43]. To filter out the most common class of non-anaphoric demonstrative noun phrases we simply discarded any matching the pattern *this... paper|study|article*.

#### Semantic features

To facilitate a robust analysis of semantic differences, we induced a "topic model" using Latent Dirichlet Analysis (LDA) [44]. LDA models each document in a corpus as a mixture of distributions over words, or "topics". For example, a topic relating to genetics will assign high probability to words such as "gene" and "DNA", a topic relating to experimental observations will prefer "rate", "time" and "effect", while a topic relating to molecular biology will highlight "transcription" and "binding". As preprocessing we divided the corpus into its constituent articles, removing stopwords and words shorter than 3 characters. We then used the MALLET toolkit [45] to induce 100 topics over the entire corpus and make a single topic assignment for each word in the corpus. We collated the predicted distribution over topics for each article in a subdomain, weighted by article word count, to produce a topic distribution for the subdomain.

An alternative perspective on semantic behaviour is provided by mapping the distribution of syntactically-informed classes of verbs across subdomains. These classes are learned by generalising over the nouns taken by verbs as subject arguments and as direct object arguments in the corpus. The learning method is a topic model similar to the LDA selectional preference model of Ó Séaghdha [46], though instead of associating each verb with a distribution over noun classes, here each noun is associated with a distribution over verb classes. The decision to study verb classes was motivated by the fact that classifications of verbs have been shown to capture a variety of important syntactic and semantic behaviour [47,48]. By learning classes directly from the corpus, we induced a classification that reflects the characteristics of biomedical text and its subdomains. For each grammatical relation considered (subject and direct object), 100 verb classes were induced and every instance of the relation in the corpus was associated with a single class.

### Measuring divergence

Our goal is to illustrate the presence or absence of significant differences among the subdomains for each feature set. The feature sets (with the exception of the sentential and discourse features) are represented as probability distributions. We therefore calculate the Jensen-Shannon divergence (JSD) [49] for each feature set between each subdomain. JSD is a finite and symmetric measurement of divergence between probability distributions, defined as

where *H *is the Shannon entropy of a distribution

JSD values range between 0 (identical distributions) and 1 (disjoint distributions).

### Random sampling for intra-subdomain divergence

Comparability of JSD values is dependent on the dimensionality of the distributions being compared: approximations of significance break down with large dimensionality [50]. Our feature sets vary widely in this respect, from 46 (POS) to over 20,000 (nouns). We therefore compute significance scores based on random sampling of the subdomains. For each subdomain, we divide its texts into units of 200 contiguous sentences, and build 101 million-word samples by drawing randomly from these units. We then calculate the pairwise JSD values between the random samples. This gives us 10,000 JSD values calculated between random articles drawn from this subdomain (hereafter called "intra-subdomain", in contrast to "inter-subdomain"). The significance of an inter-subdomain JSD value *X *between subdomains *A *and *B *is the proportion of intra-subdomain JSD values from *A *and *B *that are less than *X*. Basically, this uses the null hypothesis that the variation *between *the two subdomains is indistinguishable from random variation *within *the subdomains. Additionally, the intra-subdomain JSD values can be used by themselves to indicate how homogeneous a subdomain is with respect to the given feature set. The choice of sample size is based on general guidelines for significant corpus sizes [51], where million-word samples are considered sufficient for specialized language studies.

### Clustering

To find natural groupings of the subdomains, we perform K-means clustering directly on the distributions, using the Gap statistic [52] to choose the value for *K*. The Gap statistic uses within-cluster error and random sampling to find optimal parameters tailored to the data set. A typical measurement of within-cluster error, the sum of squared differences between objects and cluster centres, is compared with the performance on a data set randomly generated with statistical properties similar to the actual data set. As *K *increases, performance on both data sets improves, but should improve more dramatically on the actual data set as *K *approaches a natural choice for cluster count. *K *is selected as the value where the improvement in performance at *K *+ 1 is not significantly more than the improvement in performance on the random data.

### Presentation

The non-distributional sentential and discourse features are directly reported as tables. The JSD values for the lexical and syntactic feature sets are presented in four figures per feature set: a heat map, a dendrogram, a distributional line plot, and a scatter plot.

#### Heat maps

Heat maps present pairwise calculations of a metric between a set of objects: cell *< x*, *y >*is shaded according to the value of *metric*(*x*, *y*). Our heat maps show three types of values: the top half shows JSD values between pairs of subdomains. The bottom half shows the significance of the JSD values (the probability that the variation does not occur by chance), calculated as described in the section "Random sampling for intra-subdomain divergence" above. The diagonal shows the average intra-subdomain JSD value, again as described previously. In all cases, the actual values are inscribed in each square. The significance scores are shaded from white (100% significance) to black (0% significance). The JSD values are shaded from white (highest JSD value for the feature set) to black (lowest JSD value for the feature set). In other words, white indicates more absolute variation (top half) and higher significance (bottom half).

#### Dendrograms

Dendrograms present the results of hierarchical clustering performed directly on the JSD values (i.e. from the top half of the heat map). The algorithm begins with each instance (in our case, subdomains) as a singleton cluster, and repeatedly joins the two most similar clusters until all the data is clustered together. The order of these merges is recorded as a tree structure that can be visualised as a dendrogram in which the length of a branch represents the distance between its child nodes. Similarity between clusters is calculated using average cosine distance between all members, known as "average linking". The tree leaves represent data instances (subdomains) and the paths between them are proportional to the pairwise distance. This allows visualization of multiple potential clusterings, as well as a more intuitive sense of how distinct clusters truly are. Rather than choosing a set number of flat clusters, the trees mirror the nested structure of the data.

#### Distributional line plots

The line plots present the distribution of intra-subdomain JSD values, with each line representing a subdomain. Higher values for one subdomain versus another shows that its texts have more variety with respect to that feature.

#### Scatter plots

The scatter plots project the optimal K-Means clustering onto the first two principal components of the data. The components are normalised, and points coloured according to cluster membership, with the subdomain written immediately above. The "Newswire" subdomain is not included in the plots: as an outlier, it compresses the subdomains into unreadability. In clustering, it was typically grouped with "Ethics" and "Education", or its own singleton cluster.

## Results and Discussion

### General observations

**Figure 2:**
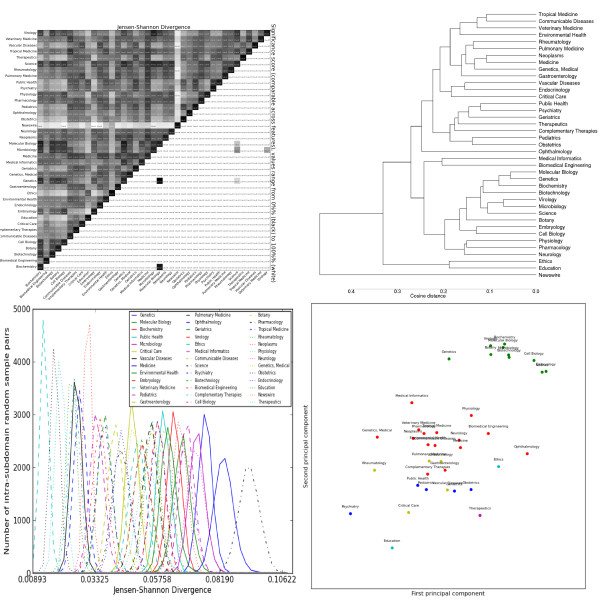
**Distributions over adjective lemmas as tagged by the C&C parser trained on Genia**. Clockwise from the top left: the heatmap shows the pairwise Jensen-Shannon Divergence (top half) and statistical significance (bottom half), as well as the homogeneity (diagonal). The dendrogram shows hierarchical clustering based on cosine difference between each subdomain's JSD values. The scatter plot is colored according to the best K-means clustering (determined by the Gap statistic) projected onto the first two principal components (normalized). The line plot shows the intra-subdomain spread of JSD values generated by random sampling.

**Figure 3:**
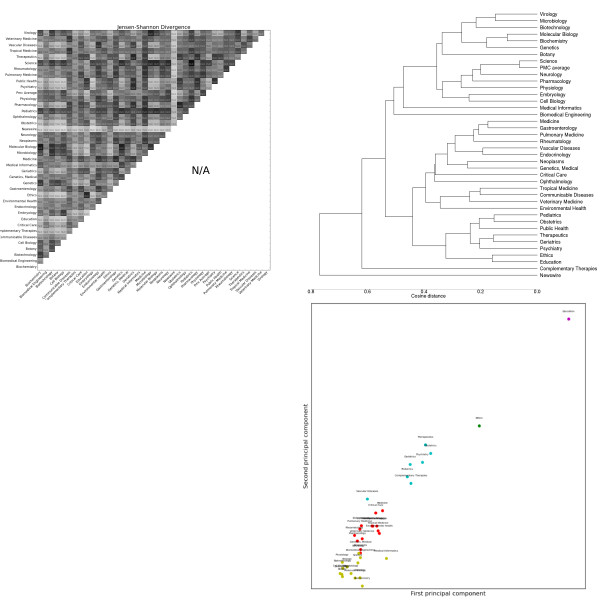
**Distributions over latent topics as modelled by Latent Dirichlet Analysis**. Clockwise from the top left: the heatmap shows the pairwise Jensen-Shannon Divergence (top half) and statistical significance (bottom half), as well as the homogeneity (diagonal). The dendrogram shows hierarchical clustering based on cosine difference between each subdomain's JSD values. The scatter plot is colored according to the best K-means clustering (determined by the Gap statistic) projected onto the first two principal components (normalized).

**Figure 4:**
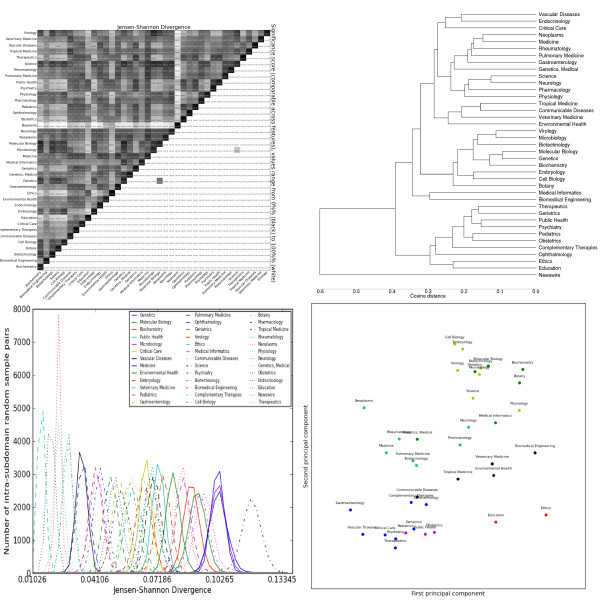
**Distributions over noun lemmas as tagged by the C&C parser trained on Genia**. Clockwise from the top left: the heatmap shows the pairwise Jensen-Shannon Divergence (top half) and statistical significance (bottom half), as well as the homogeneity (diagonal). The dendrogram shows hierarchical clustering based on cosine difference between each subdomain's JSD values. The scatter plot is colored according to the best K-means clustering (determined by the Gap statistic) projected onto the first two principal components (normalized). The line plot shows the intra-subdomain spread of JSD values generated by random sampling.

**Figure 5:**
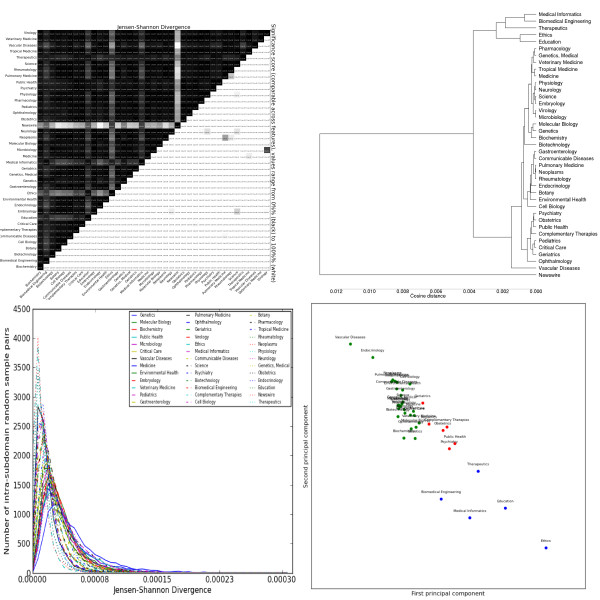
**Distributions over grammatical relations extracted by the C&C parser trained on Genia**. Clockwise from the top left: the heatmap shows the pairwise Jensen-Shannon Divergence (top half) and statistical significance (bottom half), as well as the homogeneity (diagonal). The dendrogram shows hierarchical clustering based on cosine difference between each subdomain's JSD values. The scatter plot is colored according to the best K-means clustering (determined by the Gap statistic) projected onto the first two principal components (normalized). The line plot shows the intra-subdomain spread of JSD values generated by random sampling.

The most striking general trend is the strong similarity between *Biochemistry*, *Genetics *and *Molecular Biology*. These subdomains form the most consistent cluster across feature sets, and are often one of the most closely-related triplets in the dendrograms (e.g. adjectives, Figure 2, topic modelling, Figure 3). As mentioned previously, these subdomains are the basis for most annotated resources for BioNLP. Our results suggest that not only are these resources tuned for a handful of subdomains, but these subdomains exhibit a narrow range of linguistic behaviour, less representative of other biomedical subdomains. This is true for both lexical features (e.g. nouns, Figure 4) and syntactic features (e.g. GRs, Figure 5).

The heatmaps show that, for all feature sets, the variation is significant between nearly all pairs of subdomains (exceptions are discussed below). The intra-subdomain variation is much greater and more diverse for the vocabulary features than for the POS and GR features, with the CCG features in between. The *Science *subdomain's generalist scope (encompassing journals such as *Science *and *Endeavour*) gives it unusually high intra-subdomain scores, and we don't consider it further. *Newswire *is the least similar outlier for every feature set, and is not included in the PCA plots to improve readability.

Some clusters of subdomains recur across features, and we present a useful breakdown in Table 3: these subdomain clusters are present in the optimal clustering for at least 8/10 of the feature sets. The first cluster includes subdomains dealing primarily with microscopic processes and can be further subdivided into groupings of biochemical (*Biochemistry, Genetics*) and cellular (*Cell Biology*, *Embryology*) study. The second cluster includes subdomains focused on specific anatomical systems (*Endocrinology*, *Pulmonary Medicine*). The third cluster includes subdomains focused on clinical medicine (*Psychiatry*) or specific patient-types (*Geriatrics*, *Pediatrics*). The fourth and final cluster includes subdomains focused on social and ethical aspects of medicine (*Ethics*, *Education*). This is almost always the most distant cluster from the rest of PMC and usually the closest to Newswire.

**Table 3:** Stable clusters across feature sets

***Microscopic***				
*Cellular*	*Biochemical*	*System-specific*	*Clinical*	*Social*
Cell Biology	Biochemistry	Endocrinology	Geriatrics	Ethics
Virology	Molecular Biology	Rheumatology	Pediatrics	Education
Microbiology	Genetics	Pulmonary Medicine	Psychiatry	
Embryology			Obstetrics	

Subdomains that are clustered together by at least 8/10 feature sets

### Properties of the feature sets

We now consider each feature set in terms of the significance of variation and clustering of subdomains.

#### Over- and Under-use of lexical items

Before considering the lexical feature sets, we discuss a phenomenon noticed when examining the lemmas that most characterize each cluster. We compiled lemmas with extreme log-likelihood values, indicating unusual behavior relative to the corpus average [53]. We noted that they tend to define their clusters by over- or under-use of lemmas relative to the corpus average, with some favouring one extreme or the other. This may reflect differences in how lexical items vary: for nouns, over-use tends to be characteristic because the basic objects of enquiry are often disjoint between subdomains. Conversely, common verbs that are used with subdomain-specific meanings show over- and under-use (we give examples of this in the following section). These two types of variation, the introduction of completely new nouns and the modified behaviour of common verbs, call for different adaptation techniques. For example, self-training can be used to re-estimate distributional properties of common verbs but may be less successful at handling the out-of-vocabulary effects caused by unseen nouns.

#### Lexical features

Noun distributions (Figure 4) show the highest inter-subdomain divergence. Nouns also show the most intra-subdomain variation, particularly in catch-all subdomains like *Medicine*, but also in some laboratory sciences like *Microbiology *and *Genetics*. Despite high intra-subdomain variation, only one pair of subdomains have a JSD value that is not statistically significant at the 99% level: *Genetics *and *Molecular Biology*. The k-means clusters divide the subdomains according to the over-use of nouns describing the objects focused on: some examples are clinical ("patient"), genetic ("gene"), education ("student"), oncology ("cancer"), public policy ("health"), cellular ("cell") and environmental ("exposure").

**Figure 6:**
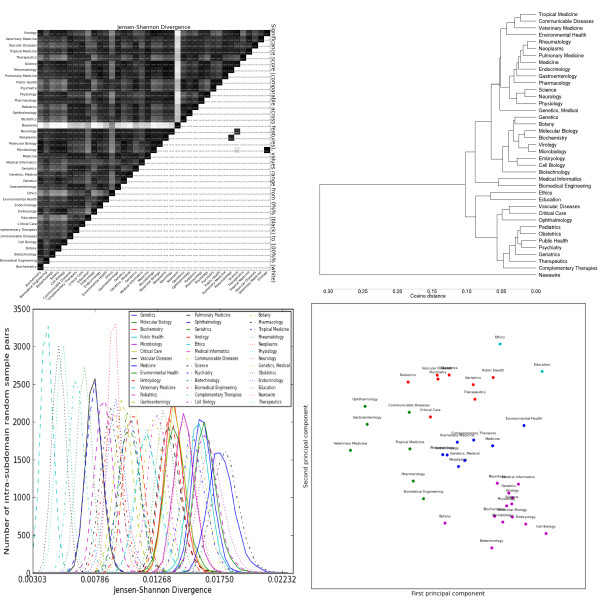
**Distributions over verb lemmas as tagged by the C&C parser trained on Genia**. Clockwise from the top left: the heatmap shows the pairwise Jensen-Shannon Divergence (top half) and statistical significance (bottom half), as well as the homogeneity (diagonal). The dendrogram shows hierarchical clustering based on cosine difference between each subdomain's JSD values. The scatter plot is colored according to the best K-means clustering (determined by the Gap statistic) projected onto the first two principal components (normalized). The line plot shows the intra-subdomain spread of JSD values generated by random sampling.

**Figure 7:**
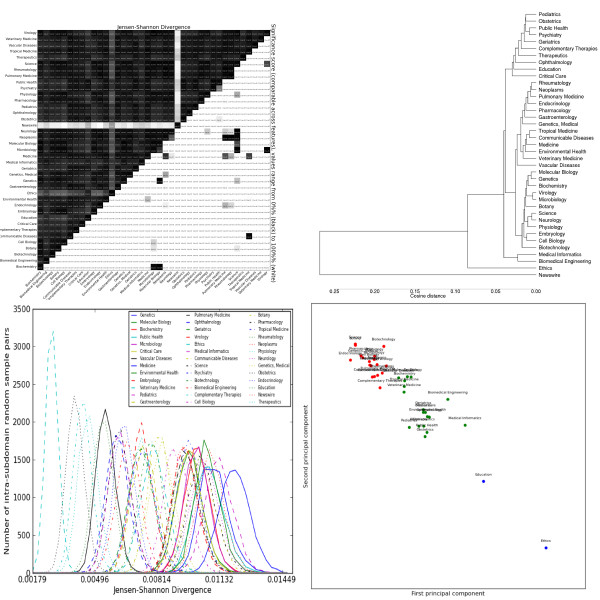
**Distributions over adverb lemmas as tagged by the C&C parser trained on Genia**. Clockwise from the top left: the heatmap shows the pairwise Jensen-Shannon Divergence (top half) and statistical significance (bottom half), as well as the homogeneity (diagonal). The dendrogram shows hierarchical clustering based on cosine difference between each subdomain's JSD values. The scatter plot is colored according to the best K-means clustering (determined by the Gap statistic) projected onto the first two principal components (normalized). The line plot shows the intra-subdomain spread of JSD values generated by random sampling.

Adjective distributions (Figure 2) also have high divergence within and between subdomains. Again, genetics-related subdomains show insignificant differences, as do *Virology *and *Microbiology*. In general, we see nouns and adjectives give common-sense semantic pairings (*Tropical Medicine *and *Communicable Disease*, *Genetics *and *Molecular Biology*) and sharply distinguish the "social sciences" from the rest. There is also a slightly less clear distinction between "patient-centric" (e.g. *Geriatrics*) and "system-centric" (e.g. *Pulmonary Medicine*) subdomains. The k-means clusters are similar to those for nouns, with the microscopic sciences (cellular and biochemical) merged into one cluster. Unlike nouns, the characteristic features include both over- and under-used terms, such as "clinical" and "medical". Verb distributions (Figure 6) have lower JSD values, but these remain significant due to lower intra-subdomain scores. The verb clusters generally agree with the noun clusters, although sometimes emphasise different similarities (e.g. *Vascular Disease *and *Critical Care *are closer together). Unlike nouns and adjectives, clusters are distinguished by both under- and over-use of verbs such as "conserve", "express" and "contain". It is also interesting that these particular verbs have specialised meanings in certain subdomains, suggesting a corresponding major shift in frequency when there is a shift in meaning. Adverbs (Figure 7) have the lowest JSD values of the lemma types. The subdomains are distinguished by two types of adverbs: markers of scientific discourse, and domain-specific premodiffers. The former include lemmas like "previously", "significantly" and "experimentally", with further distinctions between more qualitative and quantitative subdomains. The latter include lemmas like "intraperitoneally" and "immunohistochemically", which are used to avoid the more complex syntax of relative clauses and leverage the specific knowledge of its audience. These information-dense terms could prove useful for tasks like automatic curation of medical ontologies, as they imply relationships between their lexical components, the verbs they modify, and so forth.

**Figure 8:**
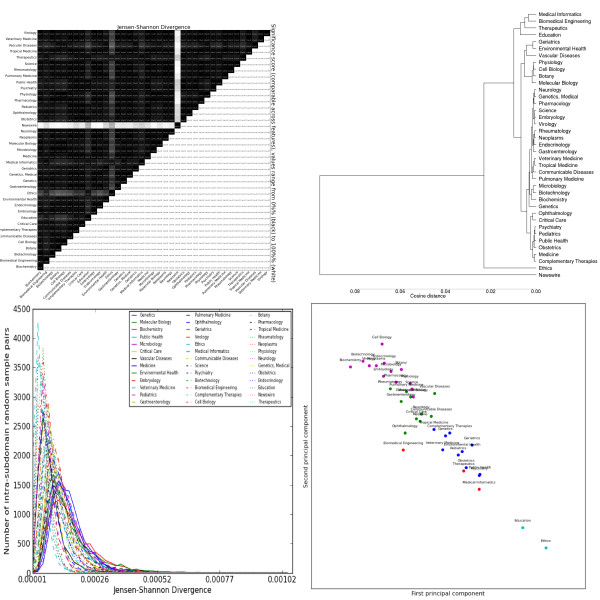
**Distributions over parts of speech as tagged by the C&C parser trained on Genia**. Clockwise from the top left: the heatmap shows the pairwise Jensen-Shannon Divergence (top half) and statistical significance (bottom half), as well as the homogeneity (diagonal). The dendrogram shows hierarchical clustering based on cosine difference between each subdomain's JSD values. The scatter plot is colored according to the best K-means clustering (determined by the Gap statistic) projected onto the first two principal components (normalized). The line plot shows the intra-subdomain spread of JSD values generated by random sampling.

POS distributions (Figure 8) have low inter-subdomain JSD values, but their even-lower intra-subdomain JSD values render them universally 100% significant. *Biomedical Engineering*, *Medical Informatics *and *Therapeutics *make particular use of present tense verbs and determiners, and markedly less of past tense. The cluster including *Communicable Disease *and *Critical Care *shows the opposite trend, perhaps reflecting certain subdomains' use of narrative. In notable contrast to the other feature sets, *Tropical Medicine *is not clustered with *Communicable Disease*, belonging instead to a cluster with distinctive overuse of comparative adjectives, foreign words and Wh-pronouns. The "laboratory science" cluster also uses many foreign words, but avoids Wh-pronouns. The difference between general language (*Newswire*), social science (*Ethics*) and the biomedical subdomains still dominates the figures. The clusters, however, are less interpretable: there are similarities with the lemma clusters, but oddities are mixed in. For example, while *Tropical Medicine*, *Communicable Disease *and *Veterinary Medicine *are still closely related, *Pulmonary Medicine *is close as well (and, as mentioned, k-means decides to split the first two, contrary to other feature sets).

#### Syntactic features

The GR features (Figure 5) have similarities with the POS features, and overlapping interpretations: for example, both capture the over-usage of determiners by *Biomedical Engineering *and *Medical Informatics*. More unusual is that their cluster includes *Ethics *and *Education*, due to high usage of clausal modifiers. This supports the claim that clauses contribute to syntactic complexity and so are typically avoided in biomedical language. It also may indicate that *Biomedical Engineering *and *Medical Informatics *retain aspects of both scientific and general language syntax. Subdomains extremely far from the centre (top-left), e.g. *Endocrinology *and *Vascular Disease*, are never grouped together in the lexical features. These outliers have particularly long average sentence length (*Vascular Disease *has the longest of all the subdomains), suggesting a relationship between GR frequencies and sentence length. However, exceptions to this (e.g. *Gastroenterology*) indicate the relationship is more complex, and requires more detailed analysis.

**Figure 9:**
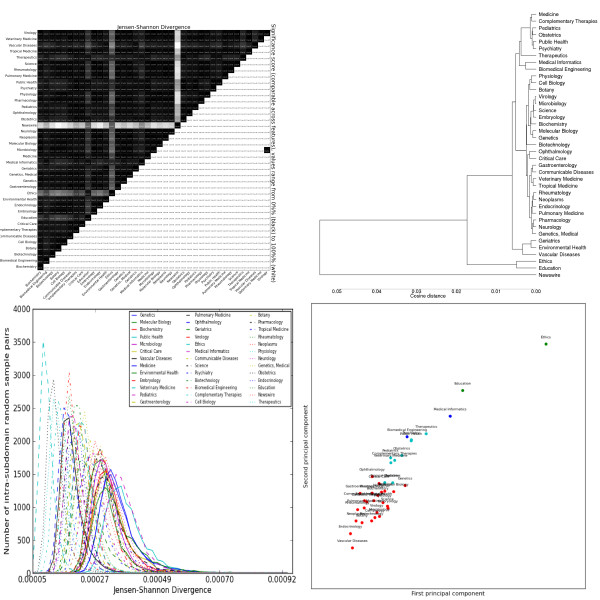
**Distributions over CCG categories extracted by the C&C parser trained on Genia**. Clockwise from the top left: the heatmap shows the pairwise Jensen-Shannon Divergence (top half) and statistical significance (bottom half), as well as the homogeneity (diagonal). The dendrogram shows hierarchical clustering based on cosine difference between each subdomain's JSD values. The scatter plot is colored according to the best K-means clustering (determined by the Gap statistic) projected onto the first two principal components (normalized). The line plot shows the intra-subdomain spread of JSD values generated by random sampling.

The CCG categories (Figure 9) show the same relationship with long sentence length as GRs. *Ethics *and *Education *are back to forming their own cluster. As mentioned previously, the distribution of intra-subdomain JSD values for CCG shows intermediate behaviour between the open-class lexical features and closed-class features, which reflects their lexical-syntactic nature. The similarities in cluster results to both the lexical and GR features demonstrates this further.

#### Sentential and discourse features

Table 4 shows each subdomain's average sentence, base noun phrase, and full noun phrase lengths (in tokens). As mentioned in the previous section, sentence length correlates with certain aspects of the syntactic features, particularly at longer sentence lengths. Without a more subtle measure of syntactic complexity we cannot say whether there is a trade-off between it and noun phrase usage, as both can lead to longer sentences. Microscopic and computational sciences, particularly *Biotechnology*, use long noun phrases. *Newswire *is towards the middle, while the social sciences form the shorter/infrequent end of the spectrum.

**Table 4:** Average sentence, base and full NP lengths (in tokens)

Subdomain	Sentence length	Average Base NP length	Average Full NP length
Vascular Diseases	28.665	1.803	3.580
Physiology	26.663	1.793	3.410
Molecular Biology	26.330	1.844	3.436
Environmental Health	26.101	1.790	3.470
Rheumatology	26.016	1.805	3.447
Biochemistry	25.981	1.846	3.569
Geriatrics	25.920	1.768	3.427
Botany	25.874	1.835	3.415
Ethics	25.842	1.655	3.172
Science	25.840	1.812	3.403
Microbiology	25.704	1.834	3.430
Tropical Medicine	25.536	1.788	3.524
Medicine	25.498	1.800	3.466
Genetics	25.433	1.827	3.424
Pulmonary Medicine	25.330	1.795	3.475
Virology	25.191	1.860	3.500
Biotechnology	25.077	1.859	3.518
Cell Biology	25.073	1.790	3.251
Neoplasms	24.983	1.849	3.467
Pharmacology	24.930	1.791	3.485
Veterinary Medicine	24.788	1.757	3.544
PMC	24.736	1.805	3.439
Public Health	24.712	1.755	3.383
Critical Care	24.611	1.802	3.471
Genetics, Medical	24.535	1.836	3.480
Psychiatry	24.482	1.752	3.412
Communicable Diseases	24.462	1.785	3.438
Embryology	24.393	1.819	3.316
Complementary Therapies	24.162	1.749	3.340
Obstetrics	24.159	1.754	3.467
Pediatrics	23.870	1.739	3.449
Gastroenterology	23.837	1.793	3.477
Education	23.653	1.719	3.303
Medical Informatics	23.579	1.785	3.365
Biomedical Engineering	23.510	1.835	3.635
Therapeutics	23.478	1.749	3.399
Neurology	23.033	1.787	3.358
Endocrinology	22.679	1.799	3.401
Newswire	19.128	1.603	3.067
Ophthalmology	17.326	1.763	3.366

Table 5 shows the frequency of pronominal/co-referential terms in each subdomain, and three finer-grained distinctions within co-reference: gendered third person (personal), neuter 3rd person (non-personal), and anaphoric determiners. Pronominal usage is highest for *Newswire*, *Ethics *and *Education*, reflecting scientific language's tendency towards explicitness. Personal pronouns are far more frequent in *Newswire *than any biomedical subdomain, due to scientific language's concern with objectivity. There is a further distinction between clinical and microscopic subdomains, in that the former are far likelier to use personal pronouns due to a focus on patients and case studies. Non-personal pronouns are frequently used in the social subdomains, in contrast to other biomedical subdomains and *Newswire*. Finally, anaphoric determiners are much less frequent in *Newswire *than any of the biomedical subdomains, and usage also increases dramatically moving from social subdomains to microscopic subdomains, where they account for over half of coreferential terms (*Virology *and *Microbiology*). This wide range (from 8% to 51%) could severely impact coreference resolution and systems that depend on it, such as information and relationship extraction.

**Table 5:** Frequency of coreferential types across domains

Subdomain	Coref. NPs		Personal		Non-personal		Anaphoric determiner	
Newswire	0.141	(227185/1614963)	0.420	(95400)	0.432	(98075)	0.084	(19121)
Ethics	0.095	(24126/252710)	0.038	(906)	0.684	(16498)	0.267	(6447)
Education	0.078	(25992/334071)	0.011	(282)	0.722	(18761)	0.262	(6810)
Medical Informatics	0.058	(318719/5516880)	0.003	(955)	0.564	(179774)	0.430	(136983)
Public Health	0.057	(182046/3207601)	0.012	(2183)	0.651	(118560)	0.332	(60395)
Therapeutics	0.054	(13928/257030)	0.009	(120)	0.640	(8919)	0.345	(4807)
Psychiatry	0.053	(26733/500995)	0.016	(429)	0.617	(16481)	0.363	(9704)
Obstetrics	0.052	(14093/270168)	0.043	(600)	0.625	(8808)	0.328	(4621)
Geriatrics	0.051	(25706/500126)	0.015	(374)	0.614	(15784)	0.368	(9469)
Genetics	0.051	(441324/8598457)	0.002	(1083)	0.499	(220079)	0.495	(218598)
Pediatrics	0.050	(17390/351237)	0.028	(488)	0.597	(10390)	0.371	(6457)
Biochemistry	0.049	(657806/13324719)	0.000	(296)	0.505	(332027)	0.493	(324072)
PMC Average	0.048	(6037808/124612679)	0.008	(49679)	0.548	(3305860)	0.441	(2660534)
Molecular Biology	0.048	(65547/1365525)	0.000	(32)	0.508	(33276)	0.490	(32098)
Tropical Medicine	0.047	(72539/1542496)	0.007	(489)	0.570	(41324)	0.421	(30519)
Critical Care	0.047	(73103/1560348)	0.008	(569)	0.570	(41637)	0.418	(30589)
Biomedical Engineering	0.046	(17502/380556)	0.003	(50)	0.527	(9224)	0.467	(8173)
Ophthalmology	0.046	(14342/313401)	0.027	(394)	0.613	(8791)	0.357	(5113)
Environmental Health	0.044	(148239/3350018)	0.009	(1323)	0.534	(79129)	0.453	(67166)
Medicine	0.044	(103490/2344444)	0.006	(666)	0.572	(59198)	0.419	(43311)
Virology	0.043	(63323/1464489)	0.002	(140)	0.490	(31057)	0.504	(31913)
Science	0.043	(470150/10903540)	0.002	(1053)	0.518	(243402)	0.477	(224195)
Rheumatology	0.043	(69365/1630635)	0.003	(181)	0.527	(36573)	0.468	(32474)
Microbiology	0.043	(129326/3042730)	0.001	(72)	0.488	(63061)	0.510	(65965)
Neurology	0.042	(82817/1967337)	0.005	(402)	0.516	(42766)	0.476	(39445)
Genetics, Medical	0.041	(29605/721049)	0.015	(452)	0.460	(13632)	0.522	(15464)
Neoplasms	0.041	(154990/3780813)	0.004	(660)	0.523	(81084)	0.471	(72947)
Communicable Diseases	0.041	(65003/1588678)	0.020	(1280)	0.497	(32286)	0.481	(31270)
Pharmacology	0.041	(15892/388714)	0.001	(15)	0.535	(8506)	0.462	(7338)
Veterinary Medicine	0.041	(21566/529841)	0.007	(145)	0.563	(12140)	0.428	(9229)
Vascular Diseases	0.041	(20669/508466)	0.004	(92)	0.565	(11684)	0.428	(8855)
Physiology	0.040	(27113/672176)	0.000	(11)	0.522	(14163)	0.474	(12862)
Embryology	0.040	(30720/767573)	0.001	(27)	0.506	(15547)	0.491	(15078)
Pulmonary Medicine	0.040	(53096/1339071)	0.002	(132)	0.551	(29245)	0.444	(23590)
Gastroenterology	0.040	(17422/440064)	0.012	(216)	0.567	(9886)	0.418	(7285)
Botany	0.039	(48611/1257981)	0.000	(19)	0.532	(25875)	0.466	(22665)
Endocrinology	0.039	(18351/476147)	0.006	(107)	0.556	(10208)	0.436	(7992)
Biotechnology	0.037	(21374/571783)	0.001	(23)	0.507	(10830)	0.490	(10475)
Cell Biology	0.037	(51864/1401952)	0.000	(17)	0.510	(26456)	0.487	(25267)
Complementary Therapies	0.025	(15558/632625)	0.008	(131)	0.673	(10467)	0.314	(4882)

#### Semantic features

A reasonable first expectation is for the topic modelling results (Figure 3) to be similar to the lexical features. This largely holds: 8/12 binary pairings of leaf subdomains in the noun dendrogram are also present in the topic modelling dendrogram, and the exceptions are generally displaced by one or two places and are attested in other vocabulary feature sets.

**Figure 10:**
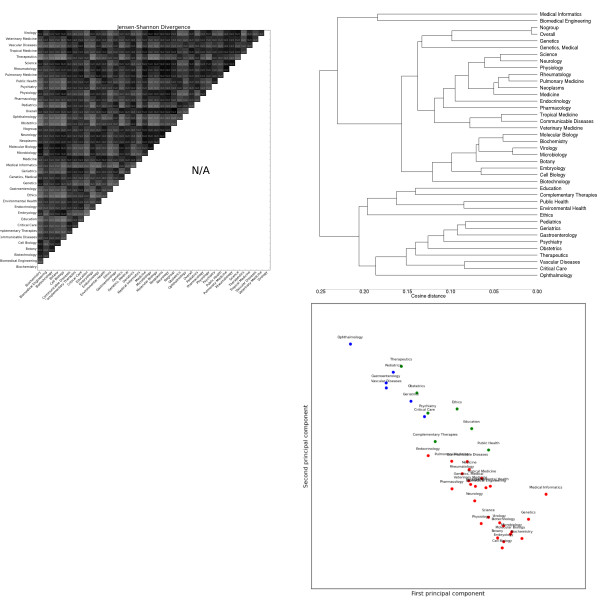
**Distributions over verb classes built from clustering on the semantics of the subject**. Clockwise from the top left: the heatmap shows the pairwise Jensen-Shannon Divergence (top half) and statistical significance (bottom half), as well as the homogeneity (diagonal). The dendrogram shows hierarchical clustering based on cosine difference between each subdomain's JSD values. The scatter plot is colored according to the best K-means clustering (determined by the Gap statistic) projected onto the first two principal components (normalized).

**Figure 11:**
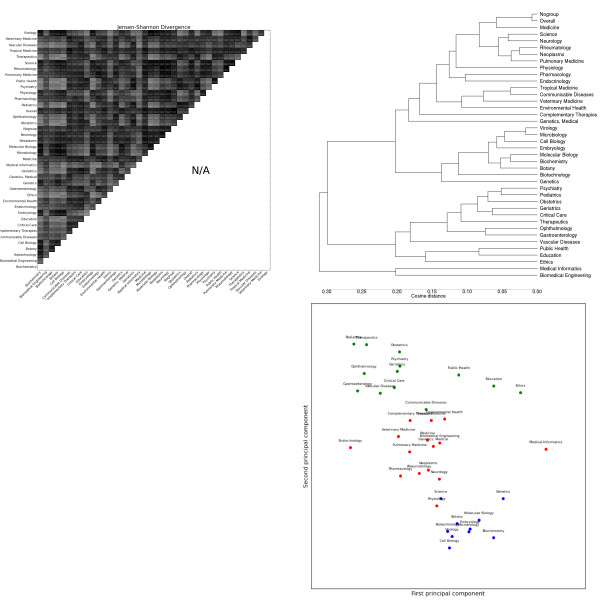
**Distributions over verb classes built from clustering on the semantics of the object**. Clockwise from the top left: the heatmap shows the pairwise Jensen-Shannon Divergence (top half) and statistical significance (bottom half), as well as the homogeneity (diagonal). The dendrogram shows hierarchical clustering based on cosine difference between each subdomain's JSD values. The scatter plot is colored according to the best K-means clustering (determined by the Gap statistic) projected onto the first two principal components (normalized).

Since the subject (Figure 10) and direct object (Figure 11) based verb cluster distributions are derived from distributions of nouns and verbs, it would be reasonable to expect them to have similarities with these feature sets. The relationship is less pronounced than between nouns and the topic models: a similar comparison of subdomain pairs has lower agreement (4/12 between nouns and both types of verb clusters, 6/12 and 7/12 between verbs and direct object-based and verbs and subject-based clusters, respectively). More significantly, the differences are often more dramatic than interpolating one or two other subdomains. For example, *Public Health *and *Psychiatry*, which are paired in the verb feature space, are distant according to both selectional preference models. Systems that use lexical frequency information at the level of syntactic arguments may need to adjust their model to account for this.

The relationship between some subdomains is stronger when considering one selectional preference versus another. For example, the similarity of *Critical Care *and *Vascular Disease *is higher for selectional preferences of subject than direct object. The reverse is true for the similarity of *Rheumatology *and *Neoplasms*. This may reflect higher usage of subdomain-specific vocabulary in particular argument positions, but this needs more in-depth scrutiny to draw detailed conclusions about selectional preferences. It does, however, show the potential importance of considering the semantics of different verbal arguments when adapting to these subdomains.

## Conclusions

In this paper we have identified the phenomenon of *subdomain variation *and studied how it manifests itself in the domain of biomedical language. As far as we are aware, this is the first time that subdomain analysis has been applied to a corpus spanning an entire scientific domain and the first time that it has been performed with a focus on implications for natural language processing applications. As well as demonstrating that subdomains do vary along many linguistic dimensions in the OpenPMC corpus, we have shown that subdomains can be clustered into relatively robust sets that remain coherent across different kinds of features. One important conclusion that directly bears on standard training and evaluation procedures for biomedical NLP tools is that the commonly-used molecular biology subdomain is not representative of the corpus as a whole and a system that performs well on a corpus from this subdomain is not guaranteed to attain comparable performance on other kinds of biomedical text. As interest in biomedical applications of NLP continues and NLP systems are deployed in increasingly varied contexts, we expect the study of subdomain variation to become ever more important. One direction for future work is to directly measure the effect of subdomain variation on the performance of NLP systems for various tasks. A second promising direction is to investigate whether system performance can be improved by integrating knowledge of the corpus' subdomain structure; a starting point for this work would be to consider Bayesian hierarchical models of the kind that have previously been suggested for modelling structural variation in a corpus [18,54].

## Authors' contributions

TL and DO collected and pre-processed the corpora and performed the feature extraction. TL carried out the distribution and clustering experiments and designed heat maps, dendrograms, plots and tables included this paper. AK contributed to the design of the experiments. All the authors took part in the analysis of the results and in the write-up of the paper. All authors read and approved this document.
